# Women’s Beliefs on Early Adherence to Adjuvant Endocrine Therapy for Breast Cancer: A Theory-Based Qualitative Study to Guide the Development of Community Pharmacist Interventions

**DOI:** 10.3390/pharmacy6020053

**Published:** 2018-06-09

**Authors:** Brittany Humphries, Stéphanie Collins, Laurence Guillaumie, Julie Lemieux, Anne Dionne, Louise Provencher, Jocelyne Moisan, Sophie Lauzier

**Affiliations:** 1 Population Health and Optimal Health Practices, CHU de Quebec–Université Laval Research Centre, Hôpital du Saint-Sacrement, 1050 chemin Ste-Foy, Quebec, QC G1S 4L8, Canada; humphrib@mcmaster.ca (B.H.); steph1collins@hotmail.com (S.C.); laurence.guillaumie@fsi.ulaval.ca (L.G.); jocelyne.moisan@pha.ulaval.ca (J.M.); 2 Faculty of Pharmacy, Université Laval, 1050 avenue de la Médecine, Quebec, QC G1V 0A6, Canada; Anne.Dionne@pha.ulaval.ca; 3 Department of Health Research Methods, Evidence and Impact, McMaster University, 1280 Main Street West, Hamilton, ON L8S 4K1, Canada; 4 Faculty of Pharmacy, Université de Montréal, 2940 Chemin de Polytechnique, Montréal, QC H3T 1J4, Canada; 5 Faculty of Nursing, Université Laval, Quebec, 1050 avenue de la Médecine, Quebec, QC G1V 0A6, Canada; 6 Oncology Research Unit, CHU de Quebec–Université Laval Research Centre, Hôpital du Saint-Sacrement, 1050 chemin Ste-Foy, Quebec, QC G1S 4L8, Canada; Julie.Lemieux@crchudequebec.ulaval.ca (J.L.); louise.provencher.cha@ssss.gouv.qc.ca (L.P.); 7 Centre des maladies du sein Deschênes-Fabia, CHU de Quebec–Université Laval, Hôpital du Saint-Sacrement, 1050 chemin Ste-Foy, Quebec, QC, G1S 4L8, Canada

**Keywords:** oncology, breast cancer, medication adherence, tamoxifen, aromatase inhibitors qualitative research

## Abstract

Adjuvant endocrine therapy (AET) taken for a minimum of five years reduces the recurrence and mortality risks among women with hormone-sensitive breast cancer. However, adherence to AET is suboptimal. To guide the development of theory-based interventions to enhance AET adherence, we conducted a study to explore beliefs regarding early adherence to AET. This qualitative study was guided by the Theory of Planned Behavior (TPB). We conducted focus groups and individual interviews among women prescribed AET in the last two years (*n* = 43). The topic guide explored attitudinal (perceived advantages and disadvantages), normative (perception of approval or disapproval), and control beliefs (barriers and facilitating factors) towards adhering to AET. Thematic analysis was conducted. Most women had a positive attitude towards AET regardless of their medication-taking behavior. The principal perceived advantage was protection against a recurrence while the principal inconvenience was side effects. Almost everyone approved of the woman taking her medication. The women mentioned facilitating factors to encourage medication-taking behaviors and cope with side effects. For adherent women, having trouble establishing a routine was their main barrier to taking medication. For non-adherent women, it was side effects affecting their quality of life. These findings could inform the development of community pharmacy-based adherence interventions.

## 1. Introduction

Adjuvant endocrine therapy (AET) (tamoxifen or aromatase inhibitors) is prescribed to women with hormone-sensitive breast cancer, approximately 75% of breast cancers [1,2]. AET has to be taken daily for five [3] or 10 years [4] to reduce the risks of recurrence and mortality. However, adherence to AET is suboptimal [5,6,7,8] as it is estimated that 28–59% of women do not take their medication on a daily basis [6,9] and 31–47% do not persist with treatment for the minimum recommended 5 years [7]. Taking <80% of AET doses has been associated with a 20% increased mortality risk [10,11]. 

Few interventions designed to enhance AET adherence have been developed and their evaluation in randomized controlled trials has provided inconclusive results [12,13,14,15,16,17]. Most of these interventions were limited to the provision of information and reminders by mail or telephone. None of the interventions systematically identified and addressed psychosocial factors from their target population [18]. In addition, baseline adherence was generally high among intervention and control groups. This limited the possibility of observing improvements in AET adherence following the intervention. Finally, no AET adherence-enhancing intervention has been developed and evaluated in community pharmacies [19]. Community pharmacies are a promising setting for this type of intervention since pharmacists have frequent encounters with women prescribed an AET. They also have the expertise to detect non-adherence and implement strategies to optimize medication use.

Identifying psychosocial factors that influence AET adherence is a crucial step in developing interventions with a potential to improve adherence. Previous qualitative studies [20,21,22,23,24,25,26,27,28,29] have furthered our understanding of the subjective experience of AET. An integrative review of the findings from these studies indicates that non-adherence to AET is multifaceted and influenced by several psychosocial factors, such as the experience of side effects, negative attitudes towards AET or medication in general, lack of a routine, and unsatisfactory relationships with healthcare professionals [30]. This review highlights how some of the factors influencing non-adherence to AET (e.g., side effects, patient-provider communication) are similar to those for non-adherence to medications prescribed for other chronic conditions [31,32]. However, other factors (e.g., fear of a breast cancer recurrence, conflicting representations about the (anti)hormonal effect of AET and the necessity of treatment) pertain specifically to the experience of AET.

Although these qualitative studies provide valuable insight into women’s experiences with AET, only one study was based on a psychosocial theory [28]. This study conducted 31 individual interviews among women with various AET-taking behaviors. It was based on the Theoretical Domains Framework, which maps constructs from many psychosocial theories [33]. The authors identified a range of barriers and facilitating factors towards AET adherence that were primarily related to the domains of beliefs about consequences, intention and goals, and behavior regulation. The relative importance of these domains varied depending on the woman’s AET-taking behavior.

There are several advantages in using psychosocial theories to inform the development and evaluation of an intervention [34]. First, they facilitate a systematic approach to identifying the potentially modifiable psychosocial factors that influence a health behavior. Second, they inform the selection of the best methods to influence these factors. This is essential to increase the potential for an effective behavioral intervention [35]. Third, they assist in the evaluation of the intervention since it is possible to describe how and to what extent the intervention affected these psychosocial factors as well as the health behavior of interest.

Among existing psychosocial theories, the Theory of Planned Behavior (TPB) has been widely used to identify the main psychosocial factors influencing health-related behaviors in quantitative [36,37,38] and qualitative studies [39]. It is considered as one of the most effective psychosocial theories to predict a behavior [36,37], such as medication adherence. The TPB postulates that an individual’s intention to adopt a behavior is influenced by three constructs: attitude, subjective norm, and perceived behavioral control [40]. Each construct is influenced by a set of beliefs that are specific to the behavior and population under study. In the context of AET adherence, these are: (1) attitudinal beliefs, which are the perceived advantages and disadvantages of taking AET as prescribed; (2) normative beliefs, which are the perception of the extent that people important to the woman approve or disapprove of her taking AET as prescribed; and (3) control beliefs, which are factors perceived to hinder or facilitate taking AET as prescribed. The TPB authors recommend documenting these beliefs among the target population using qualitative methods since the beliefs and their importance may vary depending on the behavior and population of interest [40].

Given the extent of non-adherence to AET and the lack of theory-based studies that could be used to guide the development of an adherence-enhancing intervention, we conducted a qualitative study using the TPB among women prescribed AET within the last two years. The aim was to identify women’s attitudinal, normative, and control beliefs regarding AET adherence that could be targeted by an intervention offered in the community pharmacy setting. This study was a crucial first step in addressing research gaps regarding interventions to enhance adherence to AET.

## 2. Materials and Methods

### 2.1. Design

This qualitative descriptive study was guided by the TPB. It was conducted from November 2013 to February 2014 using focus groups and individual interviews. We selected focus groups because interactions with others are likely to deepen participants’ reflections on their experience [41]. Based on the analysis of focus groups, which comprised women who were mostly adherent at the time of the study, we conducted individual interviews [42] targeting specifically non-adherent women. Individual interviews were selected because we judged that women would be more comfortable discussing this experience privately.

### 2.2. Subjects

Women were eligible to participate in the study if they were aged 18 years or older, diagnosed with hormone receptor-positive breast cancer, had a first AET prescription for early breast cancer within the last two years and sufficient fluency in French. For individual interviews, women had to self-report difficulties adhering to AET (i.e., treatment non-initiation, cessation, or suboptimal adherence on a daily basis).

Potential participants for focus group were identified by the medical team of the *Centre des maladies du sein Deschênes-Fabia* (CMS), *CHU de Québec–Université Laval*. Participants for individual interviews were identified by the CMS team, an email sent to *Université Laval* employees, and advertisements placed in Quebec City pharmacies. If a potentially eligible woman identified by the CMS team expressed interest in participating in the study, the CMS team transmitted her contact information to the research team. If the woman was informed about the study by email or advertisement, she contacted the research team herself. In both instances, a member of the research team would follow up with the woman to explain the study, verify eligibility, and solicit consent. Potential participants had no contact with the research team prior to this study.

### 2.3. Data Collection

**Figure 1:**
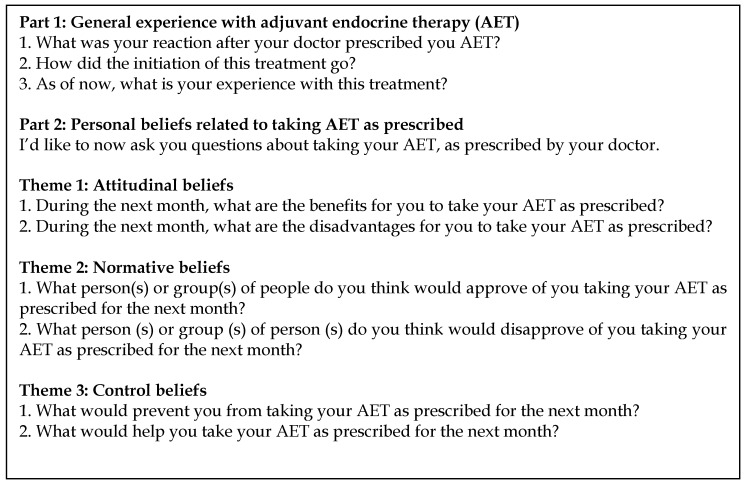
Topic guide for focus groups and individual interviews.

A topic guide comprised of questions recommended by the authors of the TPB to elicit attitudinal, normative, and control beliefs regarding AET was used for the group and individual interviews [40] (Figure 1). At the beginning of each group and individual interview, participants were reminded that researchers from *Université Laval* were interested in understanding their experience with AET. A (male) professional moderator conducted the focus groups while two members of the research team observed behind a one-way mirror (Sophie Lauzier [SL], Ph.D., Assistant Professor, trained in social and cultural anthropology and epidemiology, and Marjolaine Roy [MR], M.Sc., Research Professional, trained in psychology). The women were informed that the moderator was not part of the research team. Individual interviews were conducted by two (female) members of the research team (MR and Stephanie Collins, B.A., research assistant, trained in social and cultural anthropology). All focus group and individual interviews were tapped. In addition, each participant completed a questionnaire on their sociodemographic and treatment characteristics. They received $50 for their participation. 

### 2.4. Analysis

The principal investigator (SL) met with team members who attended the group or individual interviews to debrief after each data collection activity. Based on this preliminary analysis, it was estimated that data saturation was reached after three group and eight individual interviews. In total, five group and nine individual interviews were conducted. The transcripts were thematically analyzed [43,44] using a codebook developed through a validation process inspired by continuous thematic analysis [45]. Three team members (MR, SC, SL) developed a first version of the codebook. Codes were issued from the topic guide and also emerged from the data. The team members then independently coded transcripts from the first focus group before debriefing. This process was repeated for subsequent groups until consensus on the codebook was reached. Two team members (SC, Brittany Humphries [BH], B.A., Research Assistant, trained in social and cultural anthropology) used the final version to code remaining data, adding new codes if necessary. Codification was assisted by Microsoft WORD. Group and individual interviews were analyzed separately and then systematically compared for each code. 

### 2.5. Ethical Considerations

All participants gave informed written consent before participating in the study. The study was conducted in accordance with the Declaration of Helsinki, and the protocol was approved by the ethics committee of the *CHU de Québec*–*Université Laval* (DR-002-1425).

## 3. Results

### 3.1. Participants

For the focus groups, 72 eligible women were referred to the research team by the CMS team. Among them, 37 refused to participate and one woman could not be reached by the research team. Thus, a total of 34 women participated in five focus groups. For the individual interviews, 30 women contacted the research team. Among them, 21 were ineligible to participate. Thus, nine individual interviews were conducted (Table 1). The focus groups were conducted at *Université Laval* while the individuals interviews were conducted in a location that suited the preferences of the women (i.e., *Université Laval*, *Hôpital du Saint-Sacrement*, a medical clinic or at the woman’s home). The group and individual interviews lasted an average of 90 and 45 minutes, respectively.

### 3.2. Medication-Taking Behaviors

Focus group participants identified mainly as adherent. These women had initiated treatment, intended to persist with AET for the recommended duration and took their medication daily with few (often unintentional) missed doses. Some women had temporarily stopped taking their medication but only if recommended by a healthcare professional. Participants in the individual interviews identified to some degree as non-adherent. These women did not initiate treatment, had definitively stopped taking AET, or did not take their medication as prescribed (intentionally missing doses for days or weeks at a time).

### 3.3. Attitudinal Beliefs 

The principal perceived advantage of adhering to AET was prevention of a recurrence of cancer, although women from both the group and individual interviews understood it did not offer complete protection. Some participants expressed their fears of stopping AET, whether it was during treatment or at the end of prescription. These participants explained how taking AET afforded them a sense of security and peace of mind. In addition, several women felt a responsibility towards themselves, relatives, and their healthcare team. By not taking AET, the women felt they were betraying everyone’s efforts to get them this far.

“The advantage they told me, was that it could save me. […] I saw this as prevention against a recurrence.”

Individual Interview, Participant E.

“… All the effort made by everyone around us to support us. What’s taking a pill? We owe them that.”

Focus Group 1, Participant C.

Side effects were the main inconvenience perceived by the vast majority of participants (group and individual interviews). They ranged from hot flashes to vaginal dryness, depression, alopecia, and musculoskeletal pain. Side effects caused some women to change their daily habits (e.g., stop going to the gym because of musculoskeletal pain) and long-term goals (e.g., delay having children). Women’s experiences with other breast cancer treatments also affected how they perceived side effects. Some women who experienced side effects from other treatments affirmed they were prepared to face anything, while others rejected the possibility of feeling poorly again. Adherent women were more inclined to accommodate them. For non-adherent women, the perceived advantages of AET did not justify the inconveniences they were experiencing. Another inconvenience was how AET became a daily reminder that the women were not finished with cancer.

“If the hot flashes are to help me stay alive, then I’ll just turn down the heating system.”

Focus Group 3, Participant Z.

“It’s like a trace of what we’ve experienced, like a passport that you always have on you.”

Focus Group 4, Participant M.

### 3.4. Normative Beliefs

Although everyone approved that they take AET, most women in the group and individual interviews stated that the opinions of others did not influence any decision regarding adherence. They did, however, explain how social support helped them take their medication as prescribed. 

At the moment of prescription, the women discussed how the frantic pace of the treatments and the urgency of the situation left them with little space to reflect on what was happening. As a result, most of them relied on the judgement of their oncologist. During the treatment period, women received support from healthcare professionals such as oncologists, family doctors, oncology nurses and community pharmacists. These professionals reminded them of the benefits of AET, affirmed that the side effects they were experiencing were normal, answered their questions and informed them of the possibility of trying a different medication to reduce side effects. For women struggling with side effects, this support was a major factor in their decision to continue with treatment. Non-adherent women were more likely to have not found a healthcare professional with whom they could consult.

“Yes the doctor prescribed it to you, you trust him. It’s like chemo treatment. It’s preferable to not have it but you are told that you must.”

Focus Group 3, Participant J.

“I had tons of questions and I wasn’t able ask them because she [oncologist] was, I felt that she was anxious, busy, it seemed as though I had to go quickly. So I was left with my questions.”

Individual Interview, Participant K.

Support from relatives often came in the form of reminders. However, certain women chose not to speak of AET. Reasons include an inability to talk about AET because it references the trauma of cancer, not wanting to worry their relatives, and a desire to avoid being pitied. Several women sought out support from breast cancer survivors. Discussing this shared experience provided these women with the emotional and informational support they needed.

“I don’t talk because I’m not able to talk about it.”

Individual Interview, Participant C.

“We were twelve and twice a day, morning group and evening [discussion] group, I received all the information from them because they had gone through it before me.”

Focus Group 5, Participant S.

### 3.5. Control Beliefs

**Figure 2:**
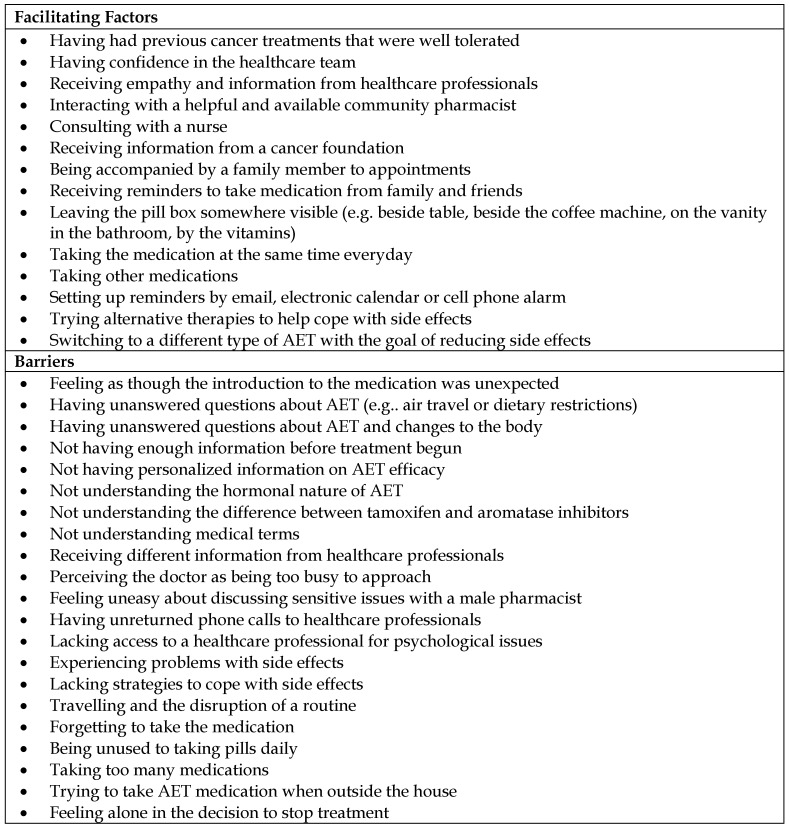
Facilitating factors and barriers to adherence to adjuvant endocrine therapy (AET).

A list of facilitating factors and barriers reported by the women is presented in Figure 2. Important facilitating factors were strategies to establish a routine. The women spoke of leaving the medication somewhere visible, taking AET at the same time, setting an alarm and using a pill box. Other facilitating factors were strategies to mitigate side effects. Several women had tried natural products, meditation, and sports while others changed the type of medication or lowered doses. Finally, women in both the group and individual interviews were unanimous in saying that obtaining answers to their questions would facilitate their adherence. To find answers, they consulted several sources of information. Participants made a distinction between blogs and “official” sources, and wanted access to scientific information in a more approachable format.

“I asked myself what do I do everyday of my life? At breakfast, my jar of peanut butter… Every morning, it is there.”

Focus Group 3, Participant F.

“She [nurse navigator] said “well, we will try another molecule. Maybe that one will serve you better than the other.” […] For me, it was like a door had opened and there could be something else that is more comfortable for five years.”

Individual Interview, Participant J.

The main barrier for adherent women was difficulty establishing a routine and forgetting their medication. For non-adherent women, it was side effects and a diminished of quality of life. A barrier common to all participants concerned the moment and quantity of information received. Most women were presented with their AET treatment plan when the pathology report was explained. Often, this information was not properly understood or retained because of the emotional charge of the meeting. In addition, women explained how their information needs varied with time. Some women had questions before they took their medication and others after they started treatment when a healthcare professional was not always accessible. Many women felt unprepared for side effects; namely that they were not warned of their severity or of potential psychological issues. Other women did not have enough information about how to incorporate AET into daily life; if they could travel by plane or eat foods containing phytoestrogens (e.g., soy, flax).

“… the hot flashes. I would wake up during the night and be drenched. I skipped one month [of AET].”

Focus Group 3, Participant F.

### 3.6. Additional Constructs

In addition to the TPB, our results indicate that the following constructs are also important for AET adherence.

#### 3.6.1. Perceived Risk

Perceived risk is an individual’s assessment of their chance to develop a health problem if they adopt a behavior [46]. Although healthcare professionals presented AET as an important preventative treatment, they often did not offer information about its efficacy specific to each woman’s medical characteristics. This was important for women suffering from side effects as it would enable them to make an informed decision about whether to continue with treatment.

“It [AET] was strongly recommended because I was in a risky age group. I had just turned 40. And then, because my cancer was hormone sensitive and very reactive…”

Individual Interview, Participant B.

#### 3.6.2. Anticipated Regret

Anticipated regret is the feeling an individual would have if the behavior was not adopted [47]. In the context of AET, several women explained that they took their medication because they would experience guilt or regret if they did not and had a recurrence.

“If I don’t take it I feel a bit guilty. I mean to say that if my cancer comes back, I’ll say well there, you didn’t follow it.”

Individual Interview, Participant H.

#### 3.6.3. Moral Standards

Moral standards are an individual’s sense of obligation towards adopting a behavior [48]. Many women felt that they had no choice but to take AET. Some felt obligated because of their role as a patient or mother, others because AET was their only adjuvant treatment.

“It is THE treatment, that’s it. I did not have anything else. I didn’t have any treatment besides this. So it was awful, but I was obligated.”

Individual Interview, Participant C.

#### 3.6.4. Self-Identity

Self-identity is the extent that an individual identifies the behavior as part of their personality [49]. In the context of AET, women indicated that character had influenced their attitude towards AET.

“I have to say that I was never a person who is very pro-medication. I was very annoyed that I had to take it.”

Individual Interview, Participant B.

## 4. Discussion

This is the first qualitative study to use the TPB to explore women’s experience with AET. Our results indicate that most women had a positive attitude towards AET regardless of their medication-taking behavior. The principal advantage perceived by participants was protection against a recurrence while the principal inconvenience was side effects. With regards to normative beliefs, almost everyone approved of the woman taking her medication and women particularly valued the support of the health care team, their relatives, and cancer survivors. The women mentioned a diversity of barriers and facilitating factors to encourage AET adherence and cope with side effects. For adherent women, having trouble establishing a routine was the main barrier to taking their medication. For non-adherent women, it was side effects and a diminished quality of life.

The main contribution of this study lies in the strength of TPB, which is considered to be one of the most effective psychosocial theories in predicting a health-related behavior [36,37]. The TPB provides a structured and comprehensive approach to investigating how three main factors (attitude, subjective norm, and perceived behavioral control) and related beliefs influence an individual’s intention and, ultimately, their health behavior. Our objective for this study was to identify women’s beliefs regarding AET adherence to guide the development of adherence-enhancing interventions . The following paragraphs will discuss our results and how they can be applied to the development of an intervention to be offered in the community pharmacy setting.

*Attitudinal beliefs*: Our findings are in line with those of previous quantitative [50,51] and qualitative studies [20,21,22,23,24,25,26,27,28]. These studies indicate that the perceived advantages of AET adherence include protection against a recurrence [21], feelings of guilt [22], and anxiety about a recurrence [25,50,51]. Interventions developed for the community pharmacy setting should therefore emphasize the advantages of taking AET medication in relation to the risk of a breast recurrence. The fact that perceived risk and anticipated regret were identified as supplementary factors in our study indicates that pharmacists should also discuss advantages related to avoiding feelings of guilt and anxiety about a recurrence. These advantages should be reiterated throughout the course of treatment to help women maintain their motivation. In addition to reinforcing the benefits of AET, pharmacists could also discuss the potential inconveniences of taking this medication. Despite the fact that most women understand the therapeutic benefits of AET, the impact of side effects on their quality of life can be so great as to outweigh any perceived advantages [20,22,50]. In a previous study, women who were informed in advance of potential side effects were more likely to be adherent [52]. Interventions should therefore address potential side effects, which were the main inconvenience identified in our study.

*Normative beliefs*: Women who participated in our study reported that the opinion of others did not directly affect their intention to be adherent to AET. They did, however, explain how social interactions influenced their medication-taking behavior. Previous studies have acknowledged that support from healthcare professionals can influence AET adherence [20,21,23,24]. In our study, women mentioned that oncologists, nurse navigators, family physicians and community pharmacists were an essential source of guidance and information. Relatives were perceived as a source of emotional and practical support while cancer survivors provided an opportunity to share the experience of taking AET with others who can understand and offer advice. Interventions for the community pharmacy setting should therefore target these three sources of social support. The intervention could raise pharmacists’ awareness of their potential to influence AET adherence. Relatives could be provided with additional resources about AET and encouraged to participate in consultations at the pharmacy, if desired by the woman. Relationships with other cancer survivors could be promoted by providing information about community-based organizations for cancer survivors and using survivors’ testimonies in the documentation given to women.

*Control beliefs:* We identified similar barriers and facilitating factors towards AET adherence as other qualitative studies [20,23,24]. These touch on information about AET, side effects and routine. With regard to information about AET, participants in our study reported that having access to timely information and answers to their questions would facilitate adherence. Information about AET was generally presented for the first time by the oncologist when pathology report was discussed. Since the retention of information may not optimal at this time, community pharmacy interventions should be designed to ensure that essential information is recapitulated when the first prescription of AET is filled. In addition, information needs vary with time. This indicates that community pharmacists should reach out to women throughout the course of treatment (e.g., at renewals). With regard to side effects, community pharmacists are at the frontline for their detection and have the potential to play an important role in helping women overcome this barrier. In our study, women who were significantly affected by side effects explained how support from healthcare professionals was an important factor in their decision to continue with treatment. Interventions should therefore ensure that community pharmacists offer some form of counselling that provides women with coping strategies. With regard to the establishment of a routine, more guidance on incorporating AET into daily life is required for some women. This includes recommendations on the timing of medication to avoid missing doses or what to do in the event of a missed dose. Pharmacists should aim to proactively detect and address practical difficulties related to taking AET on a daily basis. Their interventions could be inspired by the practical strategies mentioned by women in our study.

Our study has several strengths. First, to our knowledge, it is the first qualitative study to use the TPB to explore the experiences of women with an AET prescription. The use of the TPB contributed to our understanding of beliefs that should be targeted in a community pharmacy-based intervention aimed at enhancing AET adherence. The identification of priority targets is especially important when designing brief interventions, such as those offered in the community pharmacy setting. Second, focus groups allowed for a general portrait of the experience of women with AET while individual interviews offered a deeper understanding of non-adherence. Few of the previous qualitative studies on AET have made an explicit effort to sample women with different medication-taking behaviors [28]. This supplementary effort to meet with women who were less adherent provided unique insights into the experience of non-adherence, which can happen to varying degrees and for different reasons throughout the treatment trajectory [53].

This study also has limitations. First, our sample was restricted to women prescribed AET within the last two years. This decision was made because our team aims to develop adherence-enhancing interventions to be offered during the first years of treatment, which represents a critical opportunity to influence the entire course of treatment. However, medication-taking behaviors can change over time [53] and more theory-based qualitative research is required to understand the long-term needs of women prescribed AET. Second, we used different methods to recruit women for group and individual interviews. It is generally more difficult to recruit patients with non-adherent behaviors because they are less inclined to share their experience. Given that most of the previous qualitative studies include few non-adherent women, supplementary efforts were required to ensure that we captured this experience. We accounted for the difference between group and individual interviews in our analyses.

## 5. Conclusions

The results from this theory-based study demonstrate how qualitative data on women’s experience with AET can be used to inform the development of adherence-enhancing interventions delivered in the community pharmacy setting. Although it is within the scope of community pharmacists’ practice to inform women of advantages and inconveniences of AET (behavioral beliefs), strengthen social support (normative beliefs), and address barriers and facilitating factors (control beliefs), several considerations have to be taken into account in the design and implementation of this type of intervention. First, community pharmacists may need additional training regarding AET counselling and monitoring. A survey conducted among Canadian pharmacists indicated that they did not have sufficient knowledge or training on oral oncology treatments [54]. Second, it may be challenging for community pharmacists to perform drug monitoring throughout the 5 or 10 years of AET treatment. Studies indicate that community pharmacist’s monitoring of other long-term drug therapies is suboptimal mainly due to organizational constraints (e.g., lack of time or incentive, high workload) [55,56,57,58,59]. Given these considerations, an assessment of community pharmacists’ ability, needs and attitudes toward performing these interventions should be conducted to complement results from the present study. This would optimize the potential success of AET adherence-enhancing interventions [34].

## Reference

## Figures and Tables

**Table 1:** Sociodemographic and treatment characteristics of participants.

	Focus Groups	Individual Interviews	Total
	(*n* = 34)	(*n* = 9)	(*n* = 43)
**Age (years)**			
≤49	5	1	6
50–59	13	4	17
60–69	8	2	10
≥70	8	2	10
**Level of education**			
Primary school	1	1	2
Secondary school	7	1	8
College	11	3	14
University	15	4	19
**Time since breast cancer diagnosis (months) ^1^**			
mean (range)	16.5 (5–30)	20.3 (6–32)	17.3 (5–32)
**Breast surgery**			
Yes	34	9	43
No	0	0	0
**Other breast cancer treatments received**			
Chemotherapy	14	4	18
Radiotherapy	32	6	38
Trastuzumab	5	1	6
**Number of adjuvant treatments received in addition to AET ^1^**			
0	2	3	5
1	18	2	20
2	10	2	12
3	4	1	5
**Adjuvant endocrine treatment prescribed at time of the study**			
Tamoxifen	14	5	19
Letrozole	2	1	3
Anastrozole	18	3	21
Exemestane	0	0	0
**Time since first AET prescription (months)**			
mean (range)	10.9 (2–21)	12.6 (2–24)	11.2 (2–24)

^1^ Information not provided for *n* = 1.
